# *N*′-(3-Fluoro­benzyl­idene)-2-methyl­benzohydrazide

**DOI:** 10.1107/S1600536812025548

**Published:** 2012-06-13

**Authors:** Ying Song, Jian-Long Zhao, Jan-Gang Wang, Fei Lu, Hong Lu, Shi-Peng Li

**Affiliations:** a The 1st Affiliated Hospital of Henan University of Science & Technology, Luoyang Henan 471003, People’s Republic of China; b Medical College, Henan University of Science & Technology, Luoyang Henan 471003, People’s Republic of China

## Abstract

The asymmetric unit of the title compound, C_15_H_13_FN_2_O, contains two independent mol­ecules with different conformations; the two aromatic rings in the independent mol­ecules form dihedral angles of 85.3 (2) and 10.0 (2)°. In the crystal, N—H⋯O hydrogen bonds link the mol­ecules into chains along [100].

## Related literature

For related structures, see: Xu *et al.* (2011 ▶); Wang *et al.* (2012 ▶); Hu & Liu (2012 ▶). For the biological activity of benzohydra­zones, see: Zhang *et al.* (2012 ▶).
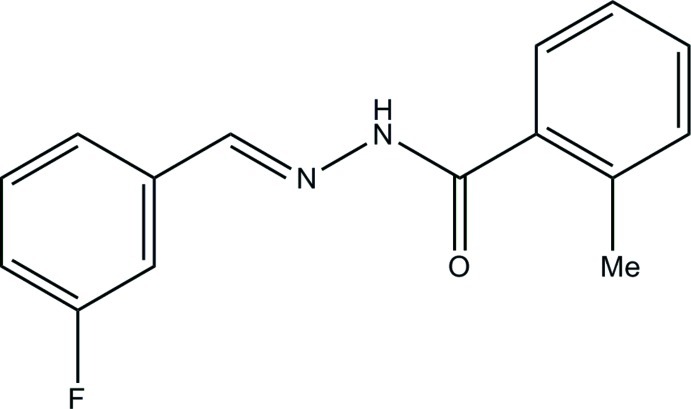


## Experimental

### 

#### Crystal data

C_15_H_13_FN_2_O
*M* *_r_* = 256.27
Triclinic, 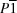

*a* = 7.8516 (13) Å
*b* = 8.1466 (13) Å
*c* = 21.158 (3) Å
α = 86.668 (2)°
β = 85.806 (2)°
γ = 79.772 (2)°
*V* = 1326.9 (4) Å^3^
*Z* = 4
Mo *K*α radiation
μ = 0.09 mm^−1^
*T* = 298 K
0.17 × 0.15 × 0.15 mm

#### Data collection

Bruker SMART CCD area-detector diffractometer
Absorption correction: multi-scan (*SADABS*; Sheldrick, 1996 ▶) *T*_min_ = 0.985, *T*_max_ = 0.986
9780 measured reflections
4844 independent reflections
4067 reflections with *I* > 2σ(*I*)
*R*_int_ = 0.019

#### Refinement

*R*[*F* ^2^ > 2σ(*F* ^2^)] = 0.041
*wR*(*F* ^2^) = 0.126
*S* = 1.06
4844 reflections
351 parameters
2 restraints
H atoms treated by a mixture of independent and constrained refinement
Δρ_max_ = 0.18 e Å^−3^
Δρ_min_ = −0.21 e Å^−3^

### 

Data collection: *SMART* (Bruker, 1998 ▶); cell refinement: *SAINT* (Bruker, 1998 ▶); data reduction: *SAINT*; program(s) used to solve structure: *SHELXS97* (Sheldrick, 2008 ▶); program(s) used to refine structure: *SHELXL97* (Sheldrick, 2008 ▶); molecular graphics: *SHELXTL* (Sheldrick, 2008 ▶); software used to prepare material for publication: *SHELXTL*.

## Supplementary Material

Crystal structure: contains datablock(s) global, I. DOI: 10.1107/S1600536812025548/cv5308sup1.cif

Structure factors: contains datablock(s) I. DOI: 10.1107/S1600536812025548/cv5308Isup2.hkl

Supplementary material file. DOI: 10.1107/S1600536812025548/cv5308Isup3.cml

Additional supplementary materials:  crystallographic information; 3D view; checkCIF report

## Figures and Tables

**Table 1:** Hydrogen-bond geometry (Å, °)

*D*—H⋯*A*	*D*—H	H⋯*A*	*D*⋯*A*	*D*—H⋯*A*
N4—H4⋯O1	0.89 (1)	1.93 (1)	2.8115 (15)	168 (2)
N2—H2⋯O2^i^	0.89 (1)	2.08 (1)	2.9185 (16)	157 (2)

Symmetry code: (i) 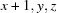.

**Fig. 1.**
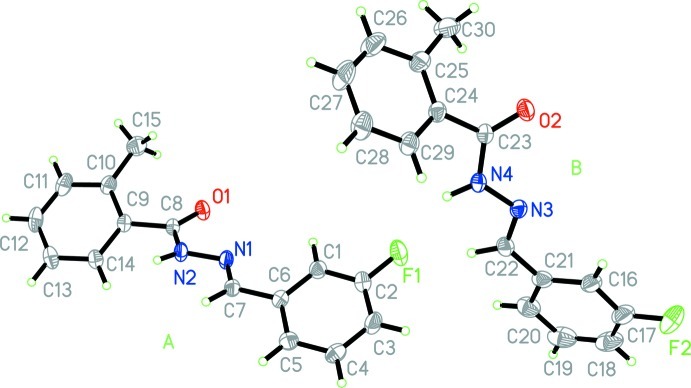
Conformations of two independent molecules in (I). Displacement ellipsoids are drawn with 30% probability level.

**Fig. 2.**
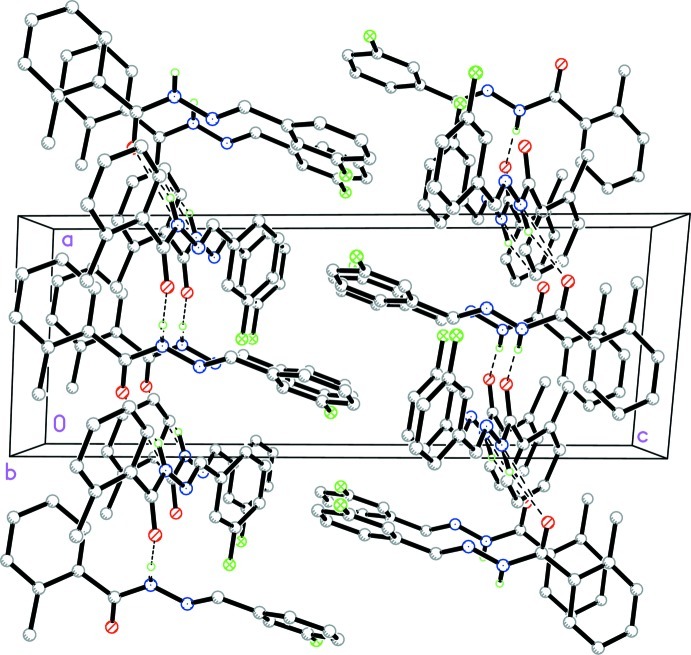
Molecular packing diagram of (I), viewed down the b axis. Hydrogen bonds are shown as thin dashed lines. C-bound H atoms omitted for clarity

## supplementary crystallographic information

### Comment

Benzohydrazones derived from the condensation reactions of benzohydrazides with carbonyl-containing compounds have been proved to have antimicrobial and antitumor activities (Zhang *et al.*, 2012). Herewith we present the title compound (I), which is a new benzohydrazone derivative.

The asymmetric unit of the title compound contains two independent molecules, A and B, respectively, with different conformations (Fig. 1) - two aromatic rings in the independent molecules form the dihedral angles of 85.3 (2)° (B) and 10.0 (2)° (A), respectively. All the bond lengths and angles are normal and correspond to those observed in the related structures (Wang *et al.*, 2012; Hu & Liu, 2012; Xu *et al.*, 2011). In the crystal structure, intermolecular N—H···O hydrogen bonds (Table 1) link the molecules into chains in [100] (Fig. 2).

### Experimental

Equimolar quantities (1 mmol each) of 3-fluorohlorobenzaldehyde and 2-methylbenzohydrazide were mixed in 50 mL methanol. The materials were stirred at room temperature for 1 h to give a clear colorless solution. Colourless block-like single crystals were obtained after a few days.

### Refinement

Atom H2 located from a Fourier map was isotropically refined, with N–H distance restrained to 0.90 (1) Å. C-bound H atoms were positioned geometrically and treated as riding on their parent atoms, with C–H distances of 0.93–0.96 Å, and with *U*_iso_(H) set to 1.2–1.5 U_eq_(C).

### Crystal data

**Table:**

C_15_H_13_FN_2_O	*Z* = 4
*M**_r_* = 256.27	*F*(000) = 536
Triclinic, *P*1	*D*_x_ = 1.283 Mg m^−^^3^
*a* = 7.8516 (13) Å	Mo *K*α radiation, λ = 0.71073 Å
*b* = 8.1466 (13) Å	Cell parameters from 5461 reflections
*c* = 21.158 (3) Å	θ = 2.6–27.0°
α = 86.668 (2)°	µ = 0.09 mm^−^^1^
β = 85.806 (2)°	*T* = 298 K
γ = 79.772 (2)°	Block, colourless
*V* = 1326.9 (4) Å^3^	0.17 × 0.15 × 0.15 mm

### Data collection

**Table:**

Bruker SMART CCD area-detector diffractometer	4844 independent reflections
Radiation source: fine-focus sealed tube	4067 reflections with *I* > 2σ(*I*)
Graphite monochromator	*R*_int_ = 0.019
ω scans	θ_max_ = 25.5°, θ_min_ = 2.6°
Absorption correction: multi-scan (*SADABS*; Sheldrick, 1996)	*h* = −9→9
*T*_min_ = 0.985, *T*_max_ = 0.986	*k* = −9→9
9780 measured reflections	*l* = −25→25

### Refinement

**Table:**

Refinement on *F*^2^	Primary atom site location: structure-invariant direct methods
Least-squares matrix: full	Secondary atom site location: difference Fourier map
*R*[*F*^2^ > 2σ(*F*^2^)] = 0.041	Hydrogen site location: inferred from neighbouring sites
*wR*(*F*^2^) = 0.126	H atoms treated by a mixture of independent and constrained refinement
*S* = 1.06	*w* = 1/[σ^2^(*F*_o_^2^) + (0.0719*P*)^2^ + 0.1885*P*] where *P* = (*F*_o_^2^ + 2*F*_c_^2^)/3
4844 reflections	(Δ/σ)_max_ = 0.001
351 parameters	Δρ_max_ = 0.18 e Å^−^^3^
2 restraints	Δρ_min_ = −0.21 e Å^−^^3^

### Special details

**Table:**

Geometry. All esds (except the esd in the dihedral angle between two l.s. planes) are estimated using the full covariance matrix. The cell esds are taken into account individually in the estimation of esds in distances, angles and torsion angles; correlations between esds in cell parameters are only used when they are defined by crystal symmetry. An approximate (isotropic) treatment of cell esds is used for estimating esds involving l.s. planes.
Refinement. Refinement of F^2^ against ALL reflections. The weighted R-factor wR and goodness of fit S are based on F^2^, conventional R-factors R are based on F, with F set to zero for negative F^2^. The threshold expression of F^2^ > 2sigma(F^2^) is used only for calculating R-factors(gt) etc. and is not relevant to the choice of reflections for refinement. R-factors based on F^2^ are statistically about twice as large as those based on F, and R- factors based on ALL data will be even larger.

### Fractional atomic coordinates and isotropic or equivalent isotropic displacement parameters (Å^2^)

**Table:**

	*x*	*y*	*z*	*U*_iso_*/*U*_eq_	
F1	0.49294 (13)	−0.42660 (13)	0.33943 (6)	0.0741 (3)	
F2	0.1795 (2)	−0.2449 (2)	0.48808 (6)	0.1094 (5)	
N1	0.86796 (15)	0.01129 (14)	0.27473 (6)	0.0424 (3)	
N2	0.95208 (15)	0.13387 (14)	0.24586 (6)	0.0432 (3)	
N3	0.38019 (14)	0.05746 (15)	0.28693 (5)	0.0409 (3)	
N4	0.46524 (15)	0.09877 (15)	0.23035 (5)	0.0419 (3)	
O1	0.70015 (12)	0.31769 (12)	0.24076 (5)	0.0480 (3)	
O2	0.27172 (13)	0.01591 (14)	0.17023 (5)	0.0523 (3)	
C1	0.72163 (19)	−0.27856 (18)	0.31576 (7)	0.0450 (3)	
H1	0.6494	−0.1987	0.2922	0.054*	
C2	0.6609 (2)	−0.41226 (19)	0.34545 (8)	0.0501 (4)	
C3	0.7605 (2)	−0.5331 (2)	0.38078 (8)	0.0591 (4)	
H3	0.7146	−0.6222	0.4004	0.071*	
C4	0.9300 (2)	−0.5191 (2)	0.38638 (8)	0.0614 (4)	
H4A	1.0003	−0.5993	0.4105	0.074*	
C5	0.9978 (2)	−0.38669 (19)	0.35649 (8)	0.0516 (4)	
H5	1.1137	−0.3798	0.3600	0.062*	
C6	0.89381 (18)	−0.26449 (17)	0.32148 (6)	0.0410 (3)	
C7	0.96683 (18)	−0.12343 (17)	0.29124 (6)	0.0417 (3)	
H7	1.0863	−0.1325	0.2841	0.050*	
C8	0.85819 (17)	0.28570 (17)	0.23110 (6)	0.0384 (3)	
C9	0.96279 (17)	0.41235 (16)	0.20355 (6)	0.0396 (3)	
C10	0.8958 (2)	0.53546 (18)	0.15847 (7)	0.0484 (4)	
C11	1.0015 (2)	0.6487 (2)	0.13572 (9)	0.0619 (5)	
H11	0.9606	0.7303	0.1053	0.074*	
C12	1.1644 (3)	0.6444 (2)	0.15654 (9)	0.0660 (5)	
H12	1.2315	0.7221	0.1401	0.079*	
C13	1.2274 (2)	0.5256 (2)	0.20142 (9)	0.0606 (4)	
H13	1.3368	0.5227	0.2160	0.073*	
C14	1.12632 (19)	0.41004 (18)	0.22478 (7)	0.0472 (3)	
H14	1.1688	0.3293	0.2553	0.057*	
C15	0.7180 (3)	0.5522 (3)	0.13424 (10)	0.0746 (6)	
H15A	0.6322	0.5901	0.1673	0.112*	
H15B	0.7008	0.4457	0.1212	0.112*	
H15C	0.7075	0.6314	0.0987	0.112*	
C16	0.2917 (2)	−0.0634 (2)	0.41323 (7)	0.0553 (4)	
H16	0.2941	−0.1400	0.3821	0.066*	
C17	0.2317 (2)	−0.0961 (3)	0.47414 (9)	0.0698 (5)	
C18	0.2250 (3)	0.0105 (3)	0.52174 (9)	0.0813 (7)	
H18	0.1828	−0.0163	0.5625	0.098*	
C19	0.2823 (3)	0.1588 (3)	0.50772 (9)	0.0807 (6)	
H19	0.2789	0.2338	0.5395	0.097*	
C20	0.3458 (2)	0.1984 (3)	0.44652 (8)	0.0649 (5)	
H20	0.3855	0.2987	0.4376	0.078*	
C21	0.34927 (18)	0.0876 (2)	0.39894 (7)	0.0477 (4)	
C22	0.41875 (18)	0.1272 (2)	0.33473 (7)	0.0469 (3)	
H22	0.4926	0.2051	0.3289	0.056*	
C23	0.40720 (17)	0.06825 (16)	0.17440 (6)	0.0397 (3)	
C24	0.52512 (18)	0.10606 (17)	0.11860 (7)	0.0424 (3)	
C25	0.4604 (2)	0.1922 (2)	0.06360 (7)	0.0541 (4)	
C26	0.5812 (3)	0.2257 (3)	0.01535 (9)	0.0756 (6)	
H26	0.5419	0.2832	−0.0215	0.091*	
C27	0.7564 (3)	0.1768 (3)	0.02029 (10)	0.0811 (6)	
H27	0.8334	0.2020	−0.0129	0.097*	
C28	0.8183 (2)	0.0910 (2)	0.07389 (9)	0.0670 (5)	
H28	0.9370	0.0567	0.0772	0.080*	
C29	0.70265 (19)	0.0561 (2)	0.12286 (8)	0.0506 (4)	
H29	0.7441	−0.0017	0.1594	0.061*	
C30	0.2710 (2)	0.2514 (3)	0.05541 (9)	0.0746 (6)	
H30A	0.2566	0.3456	0.0257	0.112*	
H30B	0.2133	0.2834	0.0955	0.112*	
H30C	0.2219	0.1629	0.0398	0.112*	
H2	1.0634 (14)	0.108 (3)	0.2319 (9)	0.080*	
H4	0.551 (2)	0.157 (2)	0.2320 (10)	0.080*	

### Atomic displacement parameters (Å^2^)

**Table:**

	*U*^11^	*U*^22^	*U*^33^	*U*^12^	*U*^13^	*U*^23^
F1	0.0524 (6)	0.0685 (6)	0.1057 (8)	−0.0265 (5)	−0.0017 (5)	0.0042 (6)
F2	0.1345 (12)	0.1237 (11)	0.0735 (8)	−0.0505 (10)	0.0027 (7)	0.0419 (8)
N1	0.0406 (6)	0.0401 (6)	0.0470 (7)	−0.0131 (5)	0.0031 (5)	0.0061 (5)
N2	0.0343 (6)	0.0405 (6)	0.0540 (7)	−0.0104 (5)	0.0045 (5)	0.0083 (5)
N3	0.0335 (6)	0.0500 (7)	0.0384 (6)	−0.0091 (5)	0.0024 (5)	0.0044 (5)
N4	0.0350 (6)	0.0532 (7)	0.0392 (6)	−0.0164 (5)	0.0043 (5)	0.0025 (5)
O1	0.0339 (5)	0.0459 (6)	0.0639 (7)	−0.0102 (4)	0.0031 (4)	0.0015 (5)
O2	0.0464 (6)	0.0652 (7)	0.0511 (6)	−0.0272 (5)	0.0032 (5)	−0.0064 (5)
C1	0.0458 (8)	0.0395 (7)	0.0492 (8)	−0.0073 (6)	−0.0031 (6)	0.0024 (6)
C2	0.0465 (8)	0.0483 (8)	0.0571 (9)	−0.0151 (7)	0.0019 (7)	−0.0028 (7)
C3	0.0700 (11)	0.0468 (9)	0.0621 (10)	−0.0209 (8)	0.0000 (8)	0.0119 (7)
C4	0.0698 (11)	0.0504 (9)	0.0622 (10)	−0.0090 (8)	−0.0134 (8)	0.0197 (8)
C5	0.0493 (9)	0.0503 (9)	0.0549 (9)	−0.0086 (7)	−0.0090 (7)	0.0067 (7)
C6	0.0449 (8)	0.0392 (7)	0.0384 (7)	−0.0082 (6)	0.0003 (6)	0.0006 (6)
C7	0.0376 (7)	0.0441 (8)	0.0433 (7)	−0.0096 (6)	−0.0001 (6)	0.0024 (6)
C8	0.0359 (7)	0.0406 (7)	0.0395 (7)	−0.0109 (6)	0.0009 (5)	0.0008 (5)
C9	0.0374 (7)	0.0371 (7)	0.0441 (7)	−0.0092 (5)	0.0051 (6)	−0.0011 (6)
C10	0.0503 (9)	0.0420 (8)	0.0506 (8)	−0.0069 (6)	0.0034 (7)	0.0050 (6)
C11	0.0706 (12)	0.0449 (9)	0.0662 (11)	−0.0111 (8)	0.0133 (9)	0.0118 (8)
C12	0.0708 (12)	0.0497 (9)	0.0805 (12)	−0.0297 (8)	0.0199 (9)	0.0004 (9)
C13	0.0490 (9)	0.0605 (10)	0.0776 (12)	−0.0259 (8)	0.0060 (8)	−0.0084 (9)
C14	0.0416 (8)	0.0456 (8)	0.0556 (9)	−0.0129 (6)	0.0016 (6)	−0.0008 (7)
C15	0.0657 (12)	0.0769 (13)	0.0791 (13)	−0.0114 (10)	−0.0182 (10)	0.0291 (10)
C16	0.0528 (9)	0.0725 (11)	0.0402 (8)	−0.0119 (8)	−0.0050 (7)	0.0075 (7)
C17	0.0599 (11)	0.0974 (14)	0.0500 (10)	−0.0156 (10)	−0.0042 (8)	0.0214 (10)
C18	0.0639 (12)	0.133 (2)	0.0398 (10)	−0.0032 (12)	0.0036 (8)	0.0069 (11)
C19	0.0677 (12)	0.1221 (19)	0.0475 (10)	0.0027 (12)	−0.0010 (9)	−0.0255 (11)
C20	0.0539 (10)	0.0865 (13)	0.0546 (10)	−0.0092 (9)	−0.0029 (8)	−0.0151 (9)
C21	0.0337 (7)	0.0681 (10)	0.0394 (8)	−0.0041 (6)	−0.0039 (6)	−0.0008 (7)
C22	0.0379 (8)	0.0583 (9)	0.0460 (8)	−0.0137 (6)	0.0001 (6)	−0.0012 (7)
C23	0.0375 (7)	0.0390 (7)	0.0429 (7)	−0.0099 (6)	0.0035 (6)	−0.0019 (6)
C24	0.0434 (8)	0.0437 (7)	0.0407 (7)	−0.0117 (6)	0.0060 (6)	−0.0054 (6)
C25	0.0562 (9)	0.0662 (10)	0.0395 (8)	−0.0121 (8)	0.0042 (7)	−0.0032 (7)
C26	0.0768 (13)	0.0993 (15)	0.0448 (10)	−0.0104 (11)	0.0115 (9)	0.0120 (9)
C27	0.0740 (13)	0.1000 (15)	0.0625 (12)	−0.0154 (11)	0.0323 (10)	0.0091 (11)
C28	0.0482 (9)	0.0761 (12)	0.0714 (12)	−0.0075 (8)	0.0223 (8)	−0.0017 (9)
C29	0.0438 (8)	0.0538 (9)	0.0518 (9)	−0.0068 (7)	0.0084 (7)	−0.0012 (7)
C30	0.0635 (12)	0.1075 (16)	0.0501 (10)	−0.0086 (11)	−0.0095 (8)	0.0069 (10)

### Geometric parameters (Å, º)

**Table:**

F1—C2	1.3598 (18)	C13—C14	1.384 (2)
F2—C17	1.357 (2)	C13—H13	0.9300
N1—C7	1.2735 (18)	C14—H14	0.9300
N1—N2	1.3824 (15)	C15—H15A	0.9600
N2—C8	1.3549 (18)	C15—H15B	0.9600
N2—H2	0.894 (9)	C15—H15C	0.9600
N3—C22	1.2689 (19)	C16—C17	1.367 (2)
N3—N4	1.3815 (15)	C16—C21	1.394 (2)
N4—C23	1.3506 (18)	C16—H16	0.9300
N4—H4	0.893 (9)	C17—C18	1.360 (3)
O1—C8	1.2263 (16)	C18—C19	1.372 (3)
O2—C23	1.2249 (16)	C18—H18	0.9300
C1—C2	1.367 (2)	C19—C20	1.394 (3)
C1—C6	1.391 (2)	C19—H19	0.9300
C1—H1	0.9300	C20—C21	1.386 (2)
C2—C3	1.366 (2)	C20—H20	0.9300
C3—C4	1.370 (3)	C21—C22	1.467 (2)
C3—H3	0.9300	C22—H22	0.9300
C4—C5	1.387 (2)	C23—C24	1.4984 (18)
C4—H4A	0.9300	C24—C29	1.389 (2)
C5—C6	1.386 (2)	C24—C25	1.404 (2)
C5—H5	0.9300	C25—C26	1.390 (2)
C6—C7	1.4675 (19)	C25—C30	1.499 (3)
C7—H7	0.9300	C26—C27	1.373 (3)
C8—C9	1.4966 (18)	C26—H26	0.9300
C9—C14	1.388 (2)	C27—C28	1.371 (3)
C9—C10	1.403 (2)	C27—H27	0.9300
C10—C11	1.390 (2)	C28—C29	1.378 (2)
C10—C15	1.503 (2)	C28—H28	0.9300
C11—C12	1.377 (3)	C29—H29	0.9300
C11—H11	0.9300	C30—H30A	0.9600
C12—C13	1.369 (3)	C30—H30B	0.9600
C12—H12	0.9300	C30—H30C	0.9600
			
C7—N1—N2	115.08 (12)	H15A—C15—H15B	109.5
C8—N2—N1	119.04 (11)	C10—C15—H15C	109.5
C8—N2—H2	120.3 (13)	H15A—C15—H15C	109.5
N1—N2—H2	120.0 (13)	H15B—C15—H15C	109.5
C22—N3—N4	114.56 (12)	C17—C16—C21	118.33 (17)
C23—N4—N3	120.56 (11)	C17—C16—H16	120.8
C23—N4—H4	121.3 (13)	C21—C16—H16	120.8
N3—N4—H4	117.7 (13)	F2—C17—C18	118.24 (17)
C2—C1—C6	118.59 (14)	F2—C17—C16	118.05 (19)
C2—C1—H1	120.7	C18—C17—C16	123.7 (2)
C6—C1—H1	120.7	C17—C18—C19	117.97 (18)
F1—C2—C3	118.08 (14)	C17—C18—H18	121.0
F1—C2—C1	118.55 (14)	C19—C18—H18	121.0
C3—C2—C1	123.37 (15)	C18—C19—C20	120.86 (19)
C2—C3—C4	117.99 (14)	C18—C19—H19	119.6
C2—C3—H3	121.0	C20—C19—H19	119.6
C4—C3—H3	121.0	C21—C20—C19	119.7 (2)
C3—C4—C5	120.65 (15)	C21—C20—H20	120.2
C3—C4—H4A	119.7	C19—C20—H20	120.2
C5—C4—H4A	119.7	C20—C21—C16	119.46 (15)
C6—C5—C4	120.37 (15)	C20—C21—C22	119.93 (16)
C6—C5—H5	119.8	C16—C21—C22	120.58 (14)
C4—C5—H5	119.8	N3—C22—C21	121.35 (13)
C5—C6—C1	119.02 (13)	N3—C22—H22	119.3
C5—C6—C7	119.63 (13)	C21—C22—H22	119.3
C1—C6—C7	121.36 (13)	O2—C23—N4	123.05 (12)
N1—C7—C6	120.57 (13)	O2—C23—C24	124.02 (13)
N1—C7—H7	119.7	N4—C23—C24	112.93 (11)
C6—C7—H7	119.7	C29—C24—C25	120.14 (13)
O1—C8—N2	122.54 (12)	C29—C24—C23	118.20 (13)
O1—C8—C9	122.65 (12)	C25—C24—C23	121.65 (13)
N2—C8—C9	114.79 (11)	C26—C25—C24	117.09 (16)
C14—C9—C10	119.96 (13)	C26—C25—C30	119.45 (16)
C14—C9—C8	118.96 (12)	C24—C25—C30	123.45 (14)
C10—C9—C8	121.05 (12)	C27—C26—C25	122.25 (18)
C11—C10—C9	117.18 (14)	C27—C26—H26	118.9
C11—C10—C15	118.83 (15)	C25—C26—H26	118.9
C9—C10—C15	123.98 (14)	C28—C27—C26	120.26 (16)
C12—C11—C10	122.50 (16)	C28—C27—H27	119.9
C12—C11—H11	118.8	C26—C27—H27	119.9
C10—C11—H11	118.8	C27—C28—C29	119.17 (17)
C13—C12—C11	119.88 (15)	C27—C28—H28	120.4
C13—C12—H12	120.1	C29—C28—H28	120.4
C11—C12—H12	120.1	C28—C29—C24	121.09 (16)
C12—C13—C14	119.22 (16)	C28—C29—H29	119.5
C12—C13—H13	120.4	C24—C29—H29	119.5
C14—C13—H13	120.4	C25—C30—H30A	109.5
C13—C14—C9	121.24 (15)	C25—C30—H30B	109.5
C13—C14—H14	119.4	H30A—C30—H30B	109.5
C9—C14—H14	119.4	C25—C30—H30C	109.5
C10—C15—H15A	109.5	H30A—C30—H30C	109.5
C10—C15—H15B	109.5	H30B—C30—H30C	109.5

### Hydrogen-bond geometry (Å, º)

**Table:**

*D*—H···*A*	*D*—H	H···*A*	*D*···*A*	*D*—H···*A*
N4—H4···O1	0.89 (1)	1.93 (1)	2.8115 (15)	168 (2)
N2—H2···O2^i^	0.89 (1)	2.08 (1)	2.9185 (16)	157 (2)

Symmetry code: (i) *x*+1, *y*, *z*.
